# Injectable, Manganese-Labeled Alginate Hydrogels as a Matrix for Longitudinal and Rapidly Retrievable 3D Cell Culture

**DOI:** 10.3390/ijms26104574

**Published:** 2025-05-10

**Authors:** Izabela Malysz-Cymborska, Dominika Golubczyk, Piotr Walczak, Luiza Stanaszek, Miroslaw Janowski

**Affiliations:** 1 Department of Neurology and Neurosurgery, School of Medicine, Collegium Medicum, University of Warmia and Mazury in Olsztyn, Warszawska 30, 10-082 Olsztyn, Poland; 2 Ti-com LLC, Władysława Trylińskiego 2, 10-001 Olsztyn, Poland; dominikagk11@gmail.com; 3 Program in Image Guided Neurointerventions, Department of Diagnostic Radiology and Nuclear Medicine, University of Maryland, 670 W. Baltimore Street, Baltimore, MD 21201, USA; pwalczak@som.umaryland.edu (P.W.); miroslaw.janowski@som.umaryland.edu (M.J.); 4 NeuroRepair Department, Mossakowski Medical Research Institute, Polish Academy of Sciences, 02-106 Warsaw, Poland; lstanaszek@imdik.pan.pl

**Keywords:** hydrogels, stem cells, 3D culture, biomaterials, cell culture, manganese, contrast agents, injectable hydrogels, cells recovery, dissolving of hydrogels

## Abstract

Hydrogels are one of the most attractive biomaterials, used in both three-dimensional (3D) and in vivo cultures. They facilitate the reconstruction of tissue microenvironments by preserving the spatial arrangement of cells, cell–cell interactions, and functional dynamics in the tissue. In this work, the long-term effect of alginate hydrogel on cell culture and the possibility of rapid cell recovery by dissolving the hydrogel were investigated. Mouse glial-restricted progenitors (GRPs) and porcine mesenchymal stem cells (MSCs) were suspended in hydrogels; their metabolic activity, viability, and expression of genes, which are involved in oxidative stress, apoptosis, proliferation, migration, and differentiation, were assessed using quantitative polymerase chain reaction (qPCR). The concentration that was able to dissolve the hydrogel and was the least harmful to the cells was 0.005 M ethylenediaminetetraacetic acid (EDTA). The metabolism of both cell types was reduced from the beginning of the experiment to day 3. From day 7 to the end of the experiment, the normalization of the GRP metabolism was observed, in contrast to the MSCs. For the apoptosis-related genes, caspase 3, 7, and B-cell leukemia (Casp3, Casp 7, Bcl2) were increased in GRPs and MSCs on days 0 and 1. After 3 and 7 days, an increase in the expression of oxidative stress genes (nuclear factor of activated T-cells 5—NFAT5 and autophagy-related 14-ATG14) was observed in cells cultured in calcium chloride (CaCl_2_). GRPs cultured in calcium alginate (CaM) were not affected and, remarkably, showed increased Antigen Kiel 67 (Ki67) levels after 30 days. In conclusion, alginate hydrogels provide an excellent environment for stem cell culture in 3D for a longer period of time, but this is dependent on the cell type. Therefore, an individual approach to cell culture is necessary, taking into account the requirements of the cells to be used.

## 1. Introduction

In vitro cell cultures have played a pivotal role in advancing medical research for over a century [1]. During these efforts, three-dimensional (3D) cell cultures emerged as a transformative innovation [2,3,4]. The mounting body of evidence supporting 3D cell cultures stems from their ability to recapitulate the complexity and intricate nature of in vivo tissues [5,6]. Achieving 3D structures involves diverse methodologies, including the use of specialized cell culture vessels [7], microfluidics [8], scaffolds [9], and hydrogels [10]. Among these, hydrogels offer a distinct advantage by facilitating the recreation of tissue microenvironments, preserving crucial aspects like spatial cellular arrangements, cell–cell interactions, and mechanical dynamics within the tissue. The specialized microenvironment supports and induces the expression of factors responsible for critical cellular processes such as adhesion, migration, proliferation, and differentiation [11]. Therefore, hydrogels find utility in both in vitro studies and as a supportive matrix for in vivo transplantations [12]. This dual role helps to mitigate the cellular stress associated with preparation and injection processes.

A variety of gelling systems have been used for cell encapsulation, with matrigel historically prominent in cancer and regenerative medicine applications [13]. However, concerns regarding tumor origin have driven the quest for clinically applicable alternatives. Hyaluronic acid-based systems have gained favor for closely mimicking the natural body microenvironment [14]. Chitosan-based solutions were initially used to support implanted cells, but recently also found utility in 3D cell cultures [15]. Silk-based hydrogels offer another promising option [16]. While these gelling systems exhibit robust performance, a significant drawback common to all is the absence of rapid, non-toxic, on-demand dissolution strategies for easy cell retrieval [17]. The dissolution of hydrogels not only facilitates stem cell culture and re-passage but also furnishes an additional tool for controlling tissue composites in vivo upon implantation.

Hydrogels based on ion cross-linkable sodium alginate offer a promising solution to this problem [18,19]. Chelating agents can effectively and quickly reverse ion-based cross-linking in these hydrogels. Moreover, alginates are known for their biocompatibility and a lack of immunogenicity. One remarkable feature of alginate hydrogels is their versatility. They can be supplemented with various factors that support vital cellular functions. Additionally, the polymerization time can be manipulated, and the hydrogels can support microscopy and other imaging modalities [20,21,22]. Thanks to this, they can be used both for in vitro rapid non-invasive screening of cell functions and drugs as well as for more sophisticated in vivo animal experiments. Alginate hydrogels show particular promise in the treatment of neurodegenerative diseases, where repairing nervous system elements is very complex and time consuming process [23,24]. It is well known that transplanted cells without adequate immunosuppressive support are rapidly rejected. Embedding these cells in a hydrogel and then implanting near the affected tissue not only enhances transplant acceptance but also helps to unlock the full potential of the cells, thus achieving a therapeutic effect. Particularly spectacular results have been observed when using alginate hydrogels for stem cell transplantation in animal models of spinal cord injuries. In adult rats with complete spinal cord transection, alginate-embedded neural stem cells (NSCs) survived, differentiated into neurons and astrocytes, and most importantly, formed synapses, improving conductivity and causing partial motor regeneration of the hind limb after eight weeks post-transplantation [25]. Similarly, 3D bioprinted alginate scaffolds containing NSCs and oligodendrocytes transplanted into injured rat spinal cords promoted motor function recovery after two months post-injury [26]. Moreover, Ghunter et al. [27] showed that alginate scaffolds with marrow stem cells (BMSCs) promoted targeted linear axonal regeneration in the injured rat spinal cords just four weeks after transplantation. However, despite the promising effects of using hydrogels in in vivo models, there remains a gap in understanding cellular processes during long-term cultivation inside alginates. Recently, we introduced an injectable, manganese-labeled alginate hydrogel formulation designed to allow the real-time monitoring of its distribution using MRI. Notably, when administered into the intrathecal space of swine, the hydrogel did not impede cerebrospinal fluid circulation, provoke inflammatory reaction, or negatively impact the animal’s health [22].

Various cell types are poised for potential clinical applications, making them invaluable candidates for testing within the alginate hydrogel system. Our research has notably focused on harnessing the potential of glial-restricted precursors (GRPs), which are challenging to access but highly potent. Indeed, these GRPs have demonstrated the remarkable ability to rescue the lifespan of dysmyelinated mice [28]. Other up-and-coming candidates are easily accessible mesenchymal stem cells (MSCs), characterized by various beneficial properties, including immunomodulation and trophic support [29]. Therefore, we selected these two highly promising cell populations to investigate their long-term behavior within alginate-based 3D cell cultures.

An essential, yet often overlooked, element of 3D cell culture is the ability to efficiently release cells from the hydrogel scaffold. This capability is crucial to facilitate cell passage, multiplication, and subsequent analysis. In view of the above, in the current study, we determined the long-term impact of alginate hydrogel on cell culture and the feasibility of using a chelating agent for rapid hydrogel dissolution to facilitate cell retrieval. To achieve this, we have used a series of readouts, including an assessment of cell viability and the expression of key genes critical to their functionality and therapeutic potential.

## 2. Results

### 2.1. Hydrogel Dissolution

**Figure 1:**
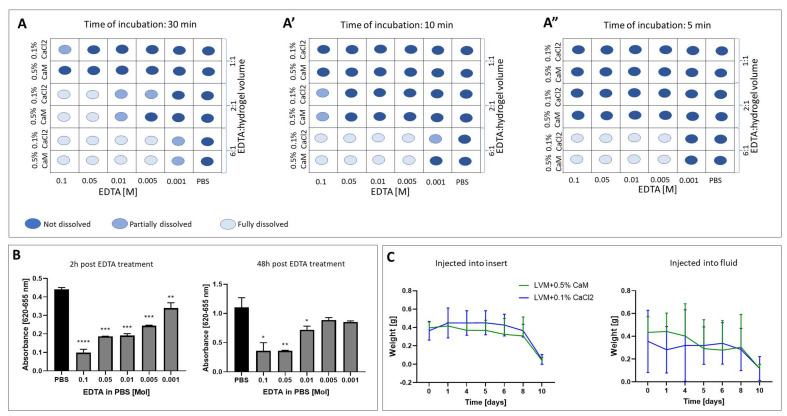
Optimization of the alginate hydrogels solutions. (**A**–**A”**) Verification of the proper percentage of solvent solution. Time of incubation in EDTA: (**A**)—30 min, (**A’**)—10 min, (**A”**)—5 min. (**B**) Analysis of the toxicity of solvents. Data are expressed as mean with standard deviation (SD) and differences were considered as statistically significant at the 95% confidence level (* *p* < 0.05, ** *p* < 0.01, *** *p* < 0.001; **** *p* < 0.0001). (**C**) Analysis of the hydrogels degradation in the fluid.

A series of ethylenediaminetetraacetic acid (EDTA) dilutions in phosphate-buffered saline (PBS) were made to test the potential of dissolving alginate hydrogels using EDTA. We used two types of hydrogels—2% low-viscosity sodium alginate (PRONOVA UP LVM; DuPont Nutrition Norge, NovaMatrix, Norway) with 1 mM manganese chloride (Sigma Aldrich, Hamburg, Germany) and LVM-MnCl_2_ + 0.1% calcium chloride (CaCl_2_) and 2% LVM-MnCl_2_ + 0.5% calcium alginate beads (CaM)—and loaded them into 12-well plates. These hydrogels were then immersed in various EDTA concentration solutions. For each solution, we conducted the experiment with three different solvent-to-hydrogel ratios: 1:1, 2:1, and 6:1. During the 30 min incubation of the hydrogels in the solvent solutions, we closely monitored their dissolution behavior. Complete dissolution was observed in 0.1 M and 0.05 M EDTA in both 2:1 and 6:1 solvent to hydrogel ratios as well as in lower percentages of EDTA—0.01 M and 0.005 M—where the solvent volume exceeded that of the hydrogel by a factor of six. In the case of EDTA solutions of 0.1 M (1:1), 0.01 M, 005 M (1:2), and 0.001 M (6:1), only a partial dissolution of the 2% LVM- + 0.1% CaCl_2_ hydrogel occurred. Similarly, 0.01 M (2:1) and 0.001 M (1:6) dissolved 2% LVM MnCl_2_ + CaM only partially (Figure 1A). Shortening the incubation time led to a reduction in the dissolving capacity of the hydrogels. The complete dissolution of hydrogels was observed in EDTA concentrations ranging from 0.1 to 0.005 M, but only when the solvent-to-hydrogel ratio was 6:1 (Figure 1A’) after 10 min of incubation. The incubation of hydrogels in 0.1 M (2:1) and 0.001 M (6:1) resulted in a partial dissolution of 2% LVM + CaCl_2_ (Figure 1A’). Moreover, after 5 min of incubation, both hydrogels completely dissolved in solutions ranging from 0.1 to 0.005 M EDTA when the solvent-to-hydrogel ratio was 6:1 (Figure 1A”).

### 2.2. Analysis of the Toxicity of Solvents

Building on the findings from the experiment, where we determined the optimal time and EDTA concentrations required for hydrogel dissolution, we proceeded to test the effect of varying solvent concentrations on cell behavior. After a 2 h period following EDTA treatment, we observed a significant decrease in cell viability (GRPs) across all EDTA dilutions, ranging from 0.1 M (*p* < 0.0001) to 0.001 M (*p* < 0.01; Figure 1A). After 48 h post-EDTA treatment, the significant decrease in cell viability was observed in wells treated with 0.1 M (*p* < 0.05), 0.05 M (*p* < 0.01), and 0.01 M (*p* < 0.05) of EDTA. Notably, no significant difference was observed between the viability of GRPs treated with 0.005 and 0.001 M and the control group (Figure 1B).

### 2.3. Evaluation of the Hydrogels Degradation

We found no statistically significant difference in the weight of the hydrogels injected into the dry insert compared to those injected directly into the fluid throughout the entire experiment (Figure 1C). In both injection methods, LVM + CaCl_2_ and LVM + CaM remained stable in terms of degradation in the fluid for a period of 6–8 days. The complete degradation of the hydrogels was observed after 10 days of incubation in PBS.

### 2.4. Hydrogel Impact on mGRP and pMSC Cell Metabolism

**Figure 2:**
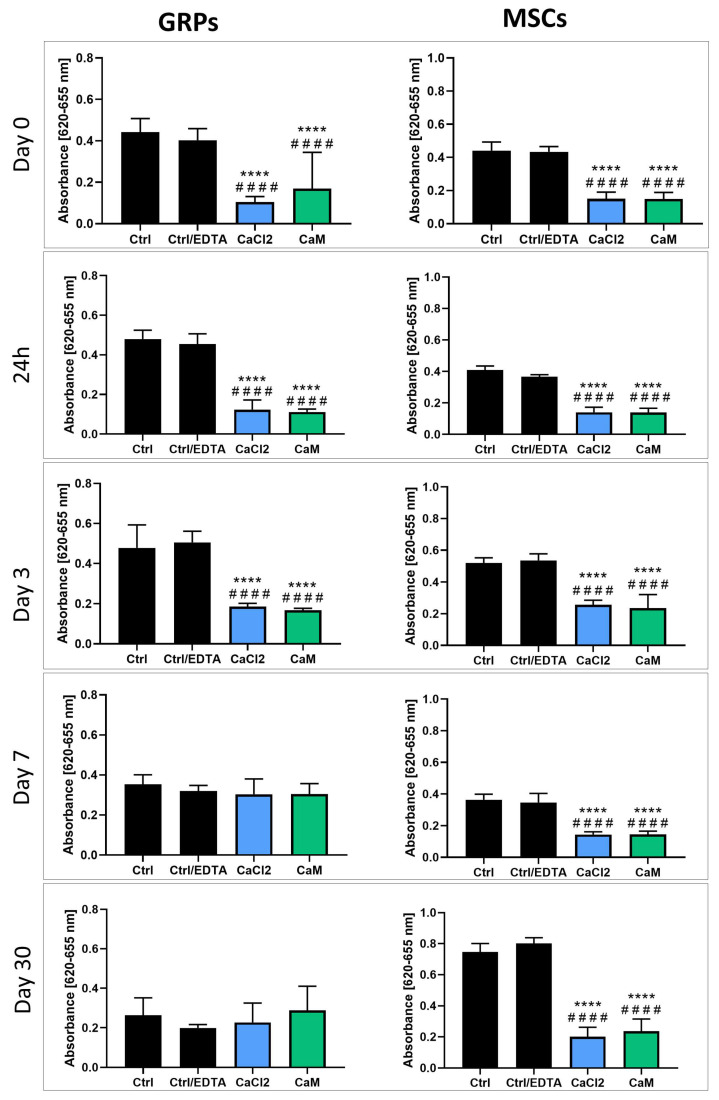
Analysis of hydrogels influence on viability of msGRP and pMSC cells—colorimetric assay. Data are expressed as mean with standard deviation (SD) and differences were considered as statistically significant at the 95% confidence level (****, #### *p* < 0.0001: *—significance in comparison with Ctrl. #—significance in comparison with Ctrl/EDTA).

The evaluation of the mGRP and pMSC metabolisms two hours after cell embedding revealed a significant decrease in metabolic activity (*p* < 0.0001) for both cell types when embedded in LVM + CaCl_2_ and LVM + CaM compared to both control groups (Figure 2). This pattern persisted after both 24 h and 3 days of incubation. Interestingly, mGRPs and pMSCs embedded in both hydrogel types showed a similar decline in metabolic rate (*p* < 0.0001) in comparison to the control groups, regardless of the presence of EDTA (Figure 2). However, after seven days of incubation, mGRPs recovered their metabolic rate, becoming indistinguishable from both controls for the remainder of the experiment (three more weeks). In contrast, the metabolic rate of pMSCs remained consistently lower when embedded in both hydrogel types, exhibiting a significant difference compared with both controls (*p* < 0.0001). After a month of cell incubation, there was no disparity in the metabolic rate of mGRPs between those embedded in hydrogels and those in the control groups. Moreover, we noticed a tendency toward an increased proliferation of mGRPs embedded in LVM + CaM hydrogels. However, the metabolic rate of pMSCs remained reduced in both hydrogel types (*p* < 0.0001) in comparison to the controls after 30 days of incubation (Figure 2).

### 2.5. Impact of Hydrogels on mGRP and pMSC Cell Viability

**Figure 3:**
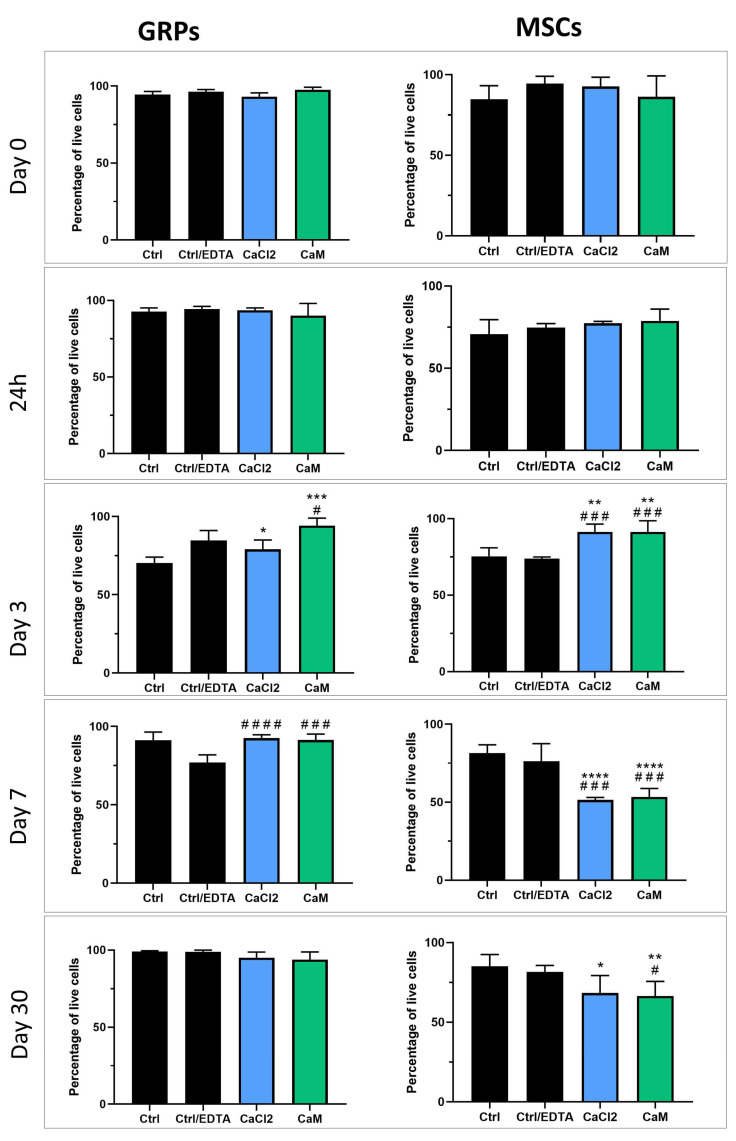
Analysis of hydrogels influence on viability of msGRP and pMSC cells—live/dead assay. Data are expressed as mean with standard deviation (SD) and differences were considered as statistically significant at the 95% confidence level (*, # *p* < 0.05, ** *p* < 0.01, ***, ### *p* < 0.001; ****, #### *p* < 0.0001: *—significance in comparison with Ctrl. #—significance in comparison with Ctrl/EDTA).

The live/dead assay results revealed a lack of a consistent negative effect from hydrogel embedment on cell viability. Specifically, at two hours post-embedding and again at 24 h, no significant differences in viability were observed between the controls and both mGRPs and pMSCs embedded in LVM + CaCl_2_ and LVM + CaM (Figure 3). Three days after cell embedding, the viability of GRPs embedded in LVM + CaCl_2_ and LVM + CaM was significantly higher compared to control (*p* < 0.05 and *p* < 0.001, respectively) and control with EDTA (LVM + CaM; *p* < 0.05). Similarly, pMSCs embedded in both hydrogel types revealed increased viability compared to control (*p* < 0.01) and ctrl/ EDTA (*p* < 0.001; Figure 3). Seven days post cell embedding, we observed an increased viability of mGRPs in both hydrogel types (*p* < 0.0001 for LVM + CaCl_2_ and LVM + CaM *p* < 0.001) compared with ctrl/EDTA. In contrast, pMSCs revealed lower viability when embedded in LVM + CaCl_2_ as well as in LVM + CaM (*p* < 0.0001) compared to control and ctrl/ EDTA (*p* < 0.001; Figure 3). The incubation of mGRPs in both types of hydrogel for a month did not result in significant changes in their viability. However, pMSCs cultured in LVM + CaCl_2_ for 30 days showed decreased viability compared to the control (*p* < 0.05). Similarly, pMSCs embedded in LVM + CaM had lower viability in comparison to the control (*p* < 0.01) and ctrl/ EDTA (*p* < 0.05; Figure 3).

### 2.6. The Impact of Extended Culture of GRPs and MSCs in Hydrogels on Gene Expression

#### 2.6.1. Oxidative Stress

**Figure 4:**
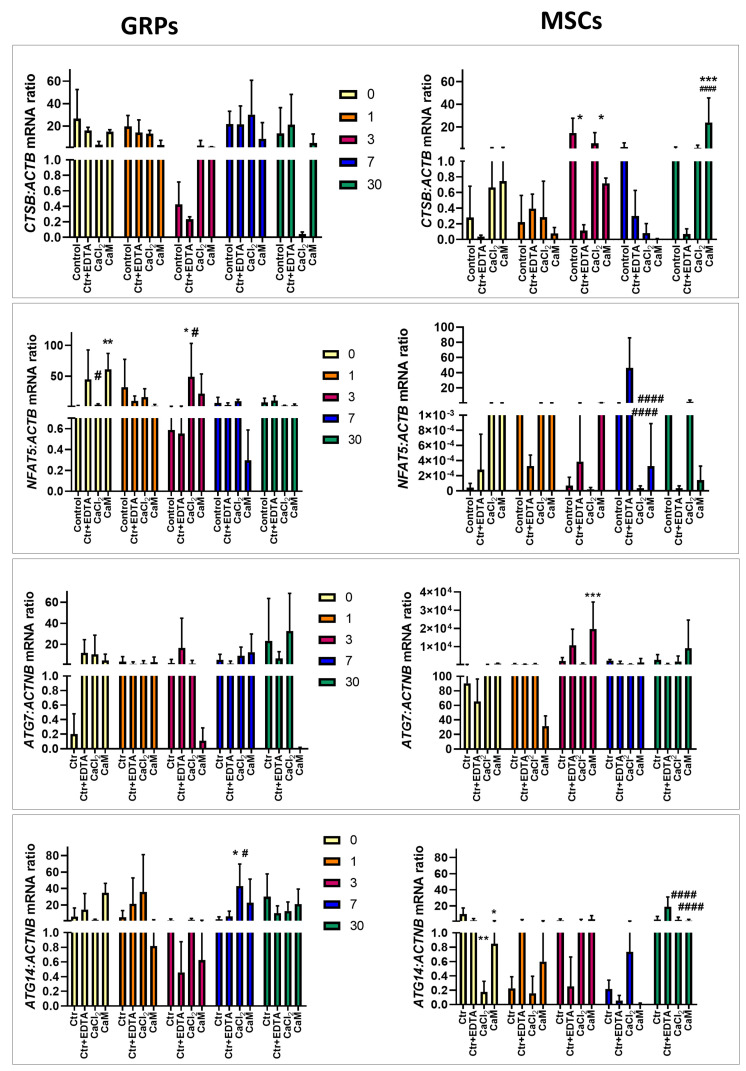
Real-time PCR analysis—effect of msGRP and pMSC cell culture in hydrogels on genes responsible for oxidative stress. Data are expressed as mean with standard deviation (SD) and differences were considered as statistically significant at the 95% confidence level (*, # *p* < 0.05, ** *p* < 0.01, ***, *p* < 0.001; #### *p* < 0.0001: *—significance in comparison with Ctrl. #—significance in comparison with Ctrl/EDTA).

Oxidative stress is a prominent factor that can occur when cultivating cells in a 3D system. To investigate this, we examined the expression of key genes associated with oxidative stress. We did not observe statistically significant changes in the expression of the cathepsin B (*CTSB*) gene in mGRP cells (Figure 4. However, we did confirm a decrease in *CTSB* expression in pMSC cultured in CaM hydrogels for 3 days compared to control (*p* < 0.05; Figure 4). In contrast, *CTSB* expression significantly increased in pMSCs cultured in CaM hydrogels for 30 days compared to control and ctrl/EDTA (*p* < 0.001; Figure 4). The nuclear factor of activated T-cells 5 (*NFAT5*) gene expression was elevated in mGRPs immediately after embedding in CaM (day 0) compared to control (*p* < 0.01; Figure 4) and decreased in mGRPs cultured in CaCL2 compared to ctrl/EDTA (*p* < 0.05; Figure 4). After 3 days post-seeding mGRPs in CaCl_2_ hydrogels, the *NFAT5* expression was significantly elevated when compared to both types of controls (*p* < 0.05; Figure 4). Interestingly, *NFAT5* expression in pMSCs was decreased after incubation for 7 days in CaCl_2_ and CaM when compared to ctrl/EDTA (*p* < 0.01; Figure 4). We did not observe any changes in autophagy-related 7 (*ATG7*) expression in mGRP cells after culturing them in both types of hydrogels throughout the experiment (Figure 4). Nevertheless, the analysis of *ATG7* expression in pMSCs showed a higher level in cells cultured in CaM for 3 days compared to control (*p* < 0.05; Figure 4). Lastly, we measured autophagy-related 14 (*ATG14)* in the context of oxidative stress. The expression of *ATG14* was elevated in GRPs cultured in CaCl_2_ hydrogels (*p* < 0.05; Figure 4). In MSCs, we observed a decrease in ATG14 expression immediately after cell embedding in both CaCl_2_ (*p* < 0.01) and CaM (*p* < 0.05) hydrogels compared to control (Figure 4). After 30 days of incubation, the level of *ATG14* was also decreased in MSCs incubated in both types of hydrogels compared to the ctrl/ EDTA (*p* < 0.001; Figure 4).

#### 2.6.2. Apoptosis

**Figure 5:**
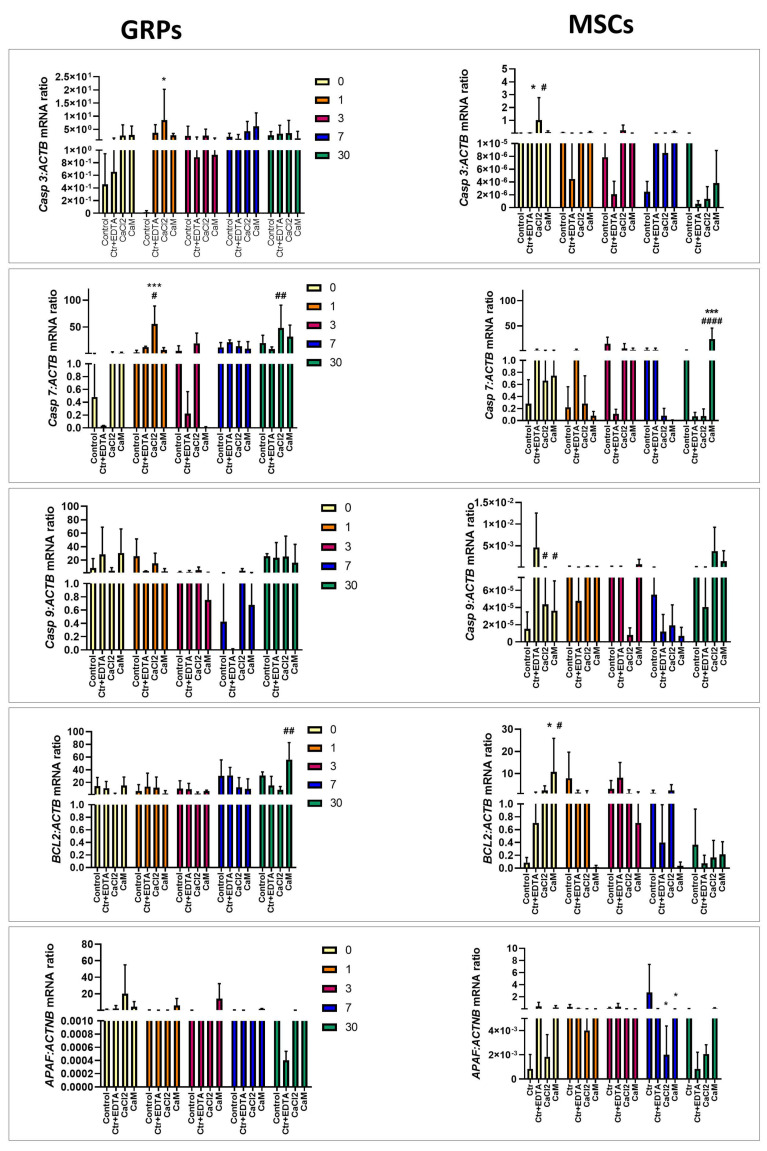
Real-time PCR analysis—effect of msGRP and pMSC cell culture in hydrogels on genes responsible for apoptosis. Data are expressed as mean with standard deviation (SD) and differences were considered as statistically significant at the 95% confidence level (*, # *p* < 0.05, ## *p* < 0.01, *** *p* < 0.001; #### *p* < 0.0001: *—significance in comparison with Ctrl. #—significance in comparison with Ctrl/EDTA).

Due to the potential induction of apoptosis resulting from prolonged exposure to various stressors on cells, our goal was to investigate whether the long-term cultivation of cells in hydrogels is associated with an alteration of crucial genes within the apoptosis pathway. Increased casapase 3 (*Casp3*) expression was observed in mGRPs cultured for 1 day in CaCl_2_ compared to control (*p* < 0.05; Figure 5) as well as in MSCs immediately post-embedding them in the CaCl_2_ and CaM (*p* < 0.05; Figure 5). The casapase 7 (*Casp7)* expression was upregulated in GRPs cultured in CaCl_2_ for 1 day compared with both types of control (*p* < 0.0001 and *p* < 0.05, respectively; see Figure 5) and in MSCs cultured for 30 days compared to control with EDTA (*p* < 0.01 Figure 5). In MSCs, we evaluated the higher expression of *Casp7* when they were cultured in CaM hydrogels for 30 days compared to ctrl/ EDTA; *p* < 0.001 and *p* < 0.0001, respectively; see Figure 5). There were no significant changes in casapase 9 (*Casp9*) expression after the mGRPs culture in both types of hydrogels (Figure 5). Upregulated *Casp9* expression was observed in MSCs immediately after embedding them as well in CaCl_2_ as in CaM (*p* < 0.05; Figure 5). The BCL2 expression was elevated in CaM-cultured GRPs after 30 days when compared to ctrl/ EDTA (*p* < 0.01; Figure 5). In the case of pMSCs, expression of *BCL2* increased immediately after embedding them in CaM hydrogels compared to both control types (*p* < 0.05; Figure 5), but the effect was rather transient. The expression of apoptotic protease-activating factor 1 (*APAF)* was changed significantly only in pMSCs cultured in CaCl_2_ and CaM hydrogels for 7 days (*p* < 0.05; Figure 5).

#### 2.6.3. Proliferation, Migration, Differentiation

**Figure 6:**
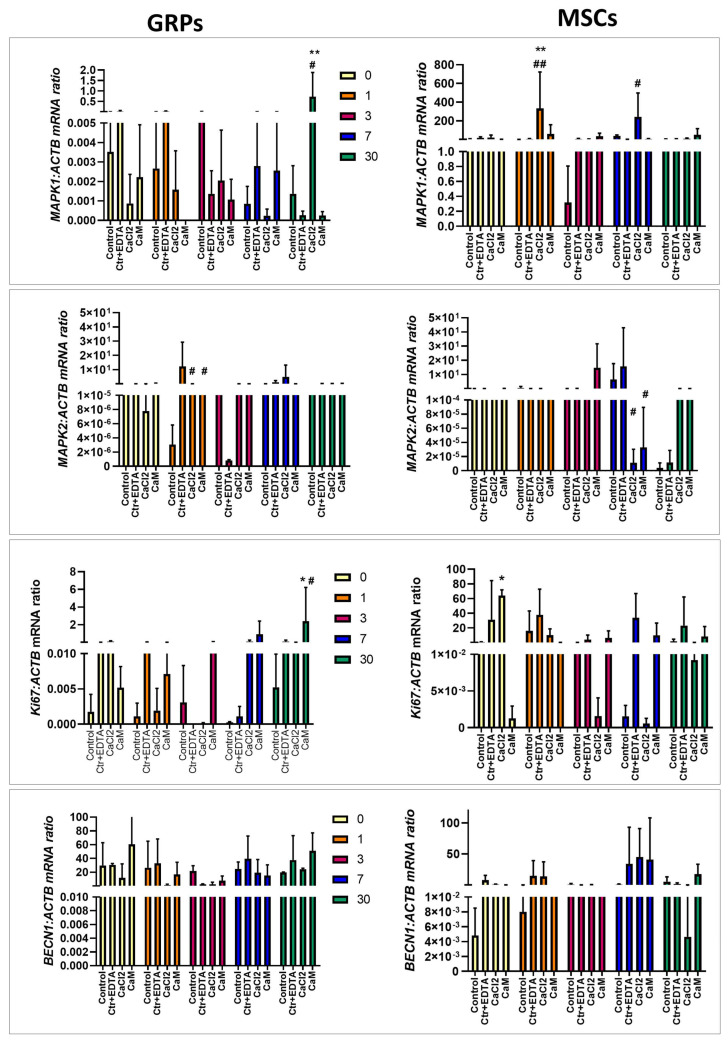
Real-time PCR analysis—effect of msGRP and pMSC cell culture in hydrogels on genes responsible for proliferation and differentiation. Data are expressed as mean with standard deviation (SD) and differences were considered as statistically significant at the 95% confidence level (*, # *p* < 0.05, **, ## *p* < 0.01: *—significance in comparison with Ctrl. #—significance in comparison with Ctrl/EDTA).

Elevated mitogen-activated protein kinase 1 (*MAPK1)* expression was observed in mGRPs cultured for 30 days in CaCl_2_ hydrogels compared to both types of control (*p* < 0.01 and *p* < 0.05, respectively; see Figure 6). Similarly, MSCs cultured in CaCl_2_ for one and seven days revealed higher *MAPK1* expression (*p* < 0.01 for one day, compared with both types of control; *p* < 0.05 for seven days when compared to ctrl/EDTA; see Figure 6). In contrast, mitogen-activated protein kinase 2 (*MAPK2)* expression decreased in mGRP cells cultured in CaCl_2_ and CaM for one day (*p* < 0.05; Figure 6). In pMSCs, lowered *MAPK2* levels were observed in cells cultured for seven days in both types of hydrogels when compared with ctrl/EDTA (*p* < 0.05; see Figure 6). The Antigen Kiel 67 (*Ki67*) expression was upregulated in CaM cultured mGRPs compared to both controls (*p* < 0.05; Figure 6). In pMSCs, *Ki67* expression increased immediately after embedding them in CaCl_2_ hydrogel compared to control (*p* < 0.05). There were no significant changes in *BECN1* expression in both types of cells after incubation in CaCl_2_ or CaM (*p* < 0.05; Figure 6).

## 3. Discussion

Previously, we extensively tested the biocompatibility and safety of alginate-based hydrogel formulas in both in vitro and in vivo settings [22]. Nevertheless, an often overlooked but essential element of cell culture in the 3D system is the ability to efficiently release cells from the hydrogel scaffold. In vitro, this capability is essential for facilitating cell passaging, expansion, and their subsequent recovery for analysis. Some analytical methods, such as gene expression analysis, may be impossible, or may yield inaccurate results when whole hydrogel scaffolds are used to isolate nucleic acids [30]. Furthermore, the utility of efficient hydrogel dissolution extends far beyond in vitro applications to in vivo settings, particularly in the context of transplantation of cell–hydrogel composites in animal models. Having a dedicated reagent for dissolving hydrogel enables swift responses to potential complications after transplantation, such as the blockage of the cerebrospinal fluid circulation or other adverse reactions to the administration of biomaterial. Given these considerations, our investigation began with the goal of identifying examining the fastest and most straight forward method for hydrogel dissolution. Knowing that EDTA has complexing properties for various cations, we decided to investigate the optimal concentration for efficiently and accurately dissolving our biomaterials. We took into account not only the effectiveness of the solvent (EDTA in PBS) but also its potential impact on the cell functionality; we simultaneously conducted tests on the solubility of the hydrogel itself and the viability of cells exposed to this dissolution process. The most effective concentration capable of dissolving the hydrogel ranged from 0.1 to 0.005 M of EDTA, with varying incubation times. Of note is that a concentration of 0.005 M EDTA, which led to complete hydrogel dissolution, proved to be the least harmful to the cells and had the least impact on cell viability.

The greatest difficulty in controlling and assessing the conditions of 3D culture is selecting an appropriate assay for evaluating viability/proliferation as well as metabolic activity. Several available, easy-to-perform commercial tests quickly allow the reading of the value of viability/proliferation and metabolic activity [31]. However, the majority of these tests were designed for standard 2D cell culture systems with little consideration for potential issues related to non-specific binding tests reagents with hydrogel components or problems with the penetration of reagents into the scaffold. Moreover, while various tests are well-suited for 2D culture settings, and are often used interchangeably, their performance in 3D culture settings can vary significantly. For this reason, we have used two viability tests to assess their performance in 3D culture settings and to learn about potential complementarity. These tests included a colorimetric [3-(4,5-dimethylthiazol-2-yl)-5-(3-carboxymethoxyphenyl)-2-(4-sulfophenyl)-2H-tetrazolium, inner salt—MTS cell titer, which is easily applicable to 3D culture settings and fluorescent live/dead assay, the latter of which requires cell extraction from the hydrogel.

We observed a significant decrease in the metabolism of GRPs in both CaCl_2_ and CaM cross-linked hydrogels from the beginning of the experiment until day 3, as indicated by the MTS assay. These results correlated with the data reported by Cao et al. [32] where the metabolism of Schwann Cells cultured in 2% alginate hydrogels decreased after 24 h, measured with MTS assay. In contrast to the colorimetric tests, the live/dead assay showed no differences between the viability of control cells and those grown in both types of hydrogels on day 0 and 24 h after. Moreover, we noticed increased viability in the post-seeding cells on days 3 and 7 in both hydrogels compared to the control, measured using live/dead assay, and an upward trend in the GRPs cultured in CaM for 30 days. This, in turn, seems to be consistent with the data of Li et al. [33], who showed that NSCs cultured for 21 days in alginate hydrogels showed much higher proliferation than cells cultured in the 2D system. Due to the similarity of the results of both tests on days 7 to 30, we believe that the differences between the measurements of MTS and live/dead assay at the beginning of the experiment may be the result of changes in the behavior and metabolism of cells in response to the shift in the environment from 2D to 3D, without affecting their viability. In the case of MSC, the metabolic activity was significantly lower in the groups of cells cultured in both 3D systems throughout the experiment. Interestingly, the viability measured by the live/dead assay showed a significant increase in MSC proliferation on day 3 from the experiment’s start in both hydrogels. Unfortunately, after 7 days and during a longer culture for 30 days, the live/dead results confirmed the data obtained from MTS, i.e., a decrease in viability in both 3D cultures. This correlates with the results of Wang et al. [34], where MSCs grown in alginate hydrogels showed slow proliferation until day 3 compared to the monolayer and then showed logarithmic growth until day 6. After this time, MSC proliferation in the hydrogels increased significantly, which can lead to their overgrowth, thus reducing the overall viability.

Being aware that using alginate hydrogels significantly alters cellular metabolism but has little impact on cell viability, we attempted to study the effect on the expression of genes critical for processes such as oxidative stress, apoptosis, or proliferation. Research on the effect of cell culture in the 3D system on gene expression has been conducted almost since the introduction of this technique [35,36,37]. It has been previously reported that a type of hydrogel and its porosity or stiffness significantly affects the expression of genes responsible for cell proliferation and differentiation [38,39]. We observed an increase in the expression *Casp3* and *Casp7*, responsible for apoptosis, mainly in the initial period (24 h) of cultivation of mGRPs in CaCl_2_ hydrogels. Additionally, *MAPK2* expression decreased on that day in mGRPs cultured in both hydrogels. In turn, there was an increase in gene expression related to oxidative stress, like *NFAT5* as well as *ATG14* in mGRPs on days *3* and *7*, coincident with the increase in proliferation in these cells exactly on these days in CaCl_2_ hydrogels. Therefore, oxidative stress, which can induce apoptosis, may act as ROS on cancer cells, increasing their proliferation and survival [40]. The stabilization of genes responsible for apoptosis and increased proliferation in mGRPs were observed after long-term culture in the *MAPK1* gene in CaCl_2_ hydrogels and *Ki67* in CaM cross-linked hydrogels. It may seem that small mGRPs adapt to it after the initial shock caused by changes in the culture environment and consequently unlock their proliferative potential. The type of cross-linker used appears essential here, as CaCl_2_ had the highest negative effect on the expression of crucial factors in mGRPs in the initial phase of culture. In the case of pMSCs, we observed an increase in the expression of genes responsible for apoptosis—*Casp3* and *Bcl2* on day 0 in the CaCl_2_ and CaM hydrogels; the increased expression of these genes was also found at the end of the experiment on day 30. Apoptosis, in this case, correlated with metabolic activity but did not correspond with changes in viability. Surprisingly, the increase in Ki67 genes on day 0 and *MAPK1* 24 h after embedding MSCs in CaCl_2_ hydrogels coincided with changes in their viability, indicating a significant increase in MSC proliferation on day 3. More interestingly, on this day, there was an increased expression of genes responsible for oxidative stress—*ATG7* and *CTSB*. After 7 and 30 days, both metabolic and viability tests and the gene expression analysis of *CTSB* and Casp7 showed decreased metabolic function and decreased MSC viability, especially in CaM cross-linked hydrogels. This, in turn, may confirm our hypothesis, consistent with the results of Wang et al. [34], about the sudden increase in the proliferation of pMSCs in alginate hydrogels after day 3 and thus the slow depletion of optimal conditions for pMSC growth in the 3D in vitro system. These 3 days of culture seem crucial for activating a cascade of events affecting the subsequent fate of the culture of these cells. It has been shown that culturing pMSCs in the 3D system for a minimum of 3 days in a spherical form increases their stemness as well as the secretion of pro-angiogenic and anti-inflammatory factors compared to 2D culture [41]. Still, on the other hand, this time point is the determinant of the in vitro conditions that are optimal for the survival of MSCs. It seems that pMSCs, due to their potential, size, and ability to proliferate rapidly, should be cultured in a 3D in vitro system for a short time, or a system should be developed to extend and improve these conditions, enabling their continuous growth. In the long run, the best setting for testing MSCs 3D composites is the in vivo system, i.e., the possibility of transplanting cell–hydrogel constructs into a living organism. As demonstrated after subcutaneous injections in rats, alginate-embedded MSCs were detected up to five weeks post-injection in contrast to non-hydrogel MSCs which were not detected after one week [42], providing evidence for excellent protection against graft rejection and allowing the slow degradation of the hydrogel and thus the possibility these cells to proliferate.

In conclusion, the findings presented in this study highlight the potential of our soluble alginate-based hydrogels as an excellent material for cultivating various stem cells in a 3D system, whether for short- or long-term applications. Nevertheless, it is important to note that the specific culture conditions and the cross-linker choice may need to be adjusted based on the size of the cells and their proliferative potential. These hydrogels possess several valuable properties including injectability, the capacity for dissolution, or the ability to be monitored in magnetic resonance imaging. These characteristics make them versatile carriers for cells, drugs, or growth factors for in vivo experiments, further enhancing their utility in regenerative medicine and tissue engineering research.

## 4. Materials and Methods

### 4.1. Experimental Design

In the initial phase of our study, we focused on optimizing the preparation and degradation of alginate hydrogels as well as the formulation of proper solvent solutions. Following this, mouse glial-restricted precursors (GRPs) and porcine mesenchymal stem cells (MSCs) were suspended in the alginate hydrogels, and we systematically assessed their metabolic activity and viability, which were measured at multiple time points. Subsequently, we delved into the impact of the alginate hydrogel system on the expression of genes involved in oxidative stress and apoptosis, as well as proliferation, migration, and differentiation. To do this, we employed quantitative polymerase chain reaction (qPCR).

### 4.2. Preparation and Dissolution of the Alginate Hydrogels

The LVM was dissolved in 4.6% mannitol (Hospira, Lake Forest, IL, USA) to form a 2% aqueous solution. The mixtures were then magnetically stirred until completely dissolved at room temperature (R.T.). Then, the manganese chloride (Sigma Aldrich, Hamburg, Germany) solution was incorporated to obtain the final concentration of 1 mM of MnCl_2_. Next, the alginate–manganese solutions were filtered over 0.22 μm filters (Greiner Bio-One, Rainbach, Austria). Subsequently, cross-linking agents, 0.1% CaCl_2_, and 0.5% solutions of calcium alginate beads (CaM, >75 μm; DuPontNutrition Norge, NovaMatrix, Sandvika, Norway) were prepared in 4.6% mannitol and sterilized in the autoclave. Next, 2% LVM was incorporated into separate syringes and connected with the syringes filled with cross-linkers using a three-way stopcock. Hydrogels were cross-linked by mixing alginate and cross-linker; they were injected via a needle into the wells of 12-well plates. Next, the hydrogels were immersed with different EDTA percentage solutions from 0.001 to 0.1 M (in PBS) and three types of volumes of the solvent: 1:1, 2:1, and 6:1 (solvent: hydrogel ratio). Hydrogels were incubated for 30 min at room temperature and then observed in the context of their solubility.

### 4.3. Spontaneous Degradation of Hydrogels in the Fluid Environment

The LVM-MnCl_2_ (4.6% mannitol) was loaded into a syringe and connected via a three-way stopcock to a syringe filled with one of two cross-linkers: either 0.1% CaCl_2_ or a 0.5% solution of CaM beads. The hydrogels were formed by mixing alginate solution with the respective cross-linker. Subsequently, they were injected via 16 G intrathecal catheter into cell strainers placed in Petri dishes. This injection was performed in two ways: into a dry cell strainer where PBS was added after 5 min, or directly into a Petri dish pre-filled with PBS. The alginate hydrogels were left to incubate at room temperature and their weight was recorded daily for a period of 10 days until complete dissolution was observed.

### 4.4. Cell Culture

Two types of stem cells were used for each assay—GRPs, with relatively low proliferative capacity, and MSCs, with much greater self-renewal potential. The porcine mesenchymal stem cells (pMSCs) were isolated as described previously [43]. Cell isolation was approved by the University of Warmia and Mazury’s local ethics committee (12/2020; 16 December 2020) and was performed according to ARRIVE guidelines. Experiments were performed according to the EU Directive 2010/63/EU as well as Poland Act 2015/01/15 “Act on the protection of animals used for scientific or educational purposes”. The bone marrow (B.M.) was aspirated from the iliac crest of the pig and mixed immediately with 10 mL of PBS (Gibco, Gaithersburg, MD, USA) with 200 μL heparin (Polfa, Warsaw, Poland). Next, B.M. was diluted in PBS in a ratio of 1:2, layered on a Ficoll-Paque Plus (1:1 ratio, Sigma Aldrich, Germany), and centrifuged at 310 g at R.T. for 25 min. Mononuclear cells were collected into a new Falcon tube with 20 mL of PBS and centrifuged at 413 g for 10 min in R.T. Next, the cell pellet was washed twice with PBS. Cells were suspended in the BMMSC medium (Gibco) and plated on 25 mL flasks at 37 °C in a humified incubator with 5% CO_2_. Cultured cells were maintained for 15–20 days (2–3 passages), harvested with Accutase (Gibco), cryopreserved in a freezing medium (Sigma Aldrich), and stored in vapor-phase liquid nitrogen for further analyses.

The mouse GRPs cells (mGRPs) were derived as described previously [44]. After this, the thawing cells were washed with PBS, centrifuged at 222 g for 5 min, plated on coated PLL/Laminin 25 mL flasks, and cultured at 37 °C in a humified incubator with 5% CO_2_. Cells were cultured for 3–5 days in GRP medium with bFGF until reaching 70% confluency, harvested with TrypLE Express (Gibco), washed with PBS, and used for the analyses.

### 4.5. Cell Metabolism Assay

To evaluate (1) the survival of mGRPs after treatment with different concentrations of EDTA as well as (2) the influence of the hydrogels’ dissolution on the pMSC and mGRP metabolisms, the cell titer 96^®^ assay (Promega, Southampton, UK) based on [3-(4,5-dimethylthiazol-2-yl)-5-(3-carboxymethoxyphenyl)-2-(4-sulfophenyl)-2H-tetrazolium, inner salt—MTS was used.

(1) The mGRPs were seeded onto 96-well plates, coated with poly-L-lysine/Laminin at a concentration of 2 × 10^4^ cells/50 μL, and cultured for 48 h in a GRP culture medium. Next, the medium was removed, and the cells were washed with PBS and immersed in a solution containing EDTA (0.001 to 0.1 M) for 5 min. After removing the EDTA, the medium with cell titer reagent (20 μL of cell titer per 100 μL medium) was added into each well and incubated for 2 h at 37 °C and 5% CO_2_. Next, the conversion of MTS into aqueous, soluble formazan was measured by absorbance at 620–665 nm wavelength using a Thermofisher Multiscan Go plate reader.

(2) The mGRPs and pMSCs were cultured on 96-well plates, one for each time point (Thermo Fischer Scientific, Waltham, MA, USA) in four experimental groups: 1—cultured as a monolayer (control); 2—cultured as a monolayer + EDTA (control with EDTA; ctrl/EDTA) treatment; 3—embedded in LVM 2% + 0.1% CaCl_2_; and 4—embedded in LVM 2% + 0.5% CaM for 2 h (day 0), 24 h, and 3, 7, and 30 days. The cells grown in hydrogels were embedded in 50 µL of the hydrogel by adding cells into 2% LVM, loading into the syringe, and cross-linking with 0.1% CaCl_2_ (3) or 0.5% CaM (4). After cross-linking, the cell–hydrogel composites were immersed with culture media and cultured at 37 °C and 5% CO_2_ (GRP or MSC, as described above). Cells from groups 1 and 2 were suspended in 100 µL GRP/MSC medium. After incubation for 2 h, 24 h, 3 days, 7 days, and 30 days, cells were washed in PBS, and 20 µL of cell titer per 100 µL culture media was added. After 2 h of incubation, the absorbance was measured as described above.

### 4.6. Live/Dead Assay

The cytotoxicity of hydrogel dissolution was assessed by the live/dead assay with Calcein AM and Ethidium homodimer-1 (ThermoFisher Scientific, Dreieich, Germany). The cells were cultured as described above. At each time interval, 2 h, 24 h, 3 days, 7 days, and 30 days post-seeding, the culture medium was removed, and the cells were washed with PBS and treated with EDTA for 5 min to dissolve the hydrogel (cells from groups 3 and 4). EDTA was then removed, and 200 µL medium with 0.05% solution of Calcein AM in PBS and EtBr (0.2% in PBS) was added per well and incubated in 37 °C and 5% CO_2_ for 30 min. Then, the solution was removed, and the cells were washed with PBS and imaged under a fluorescent microscope (Zeiss AxioVision, Oberkochen, Germany).

### 4.7. Total RNA Extraction and Reverse Transcription

To evaluate the effect of hydrogels and the dissolution procedure on the expression of crucial genes, the mGRPs and pMSCs were cultured in four experimental groups, as described in paragraphs 2.3 and 2.4. At each time interval—2 h, 24 h, and 3, 7, and 30 days post-seeding—cells were washed with PBS, treated with EDTA for 5 min (cells from groups 1–3), washed, centrifuged at 222 g for GRPs and 310 g for MSCs, collected as dry pellets, and protected at −80° for further analyses. Cells were then used for total RNA extraction using a commercial kit (A&A Biotechnology, Gdynia, Poland). RNA quality and concentration were measured using a NanoDrop 1000 spectrophotometer (Thermo Fisher Scientific Inc., Wilmington, DE, USA). Subsequently, reverse transcription reactions were carried out using the Reverse Transcription System Kit (Applied Biosystems, Foster City, CA, USA). Two types of R.T. controls were used, one without RNA and another in the absence of the reverse transcriptase.

### 4.8. Real-Time Polymerase Chain Reaction (Real-Time PCR)

The cDNA obtained was used for real-time quantitative PCR analysis using the Light Cycler 480 System (Roche, Basel, Switzerland). Each sample contained 3 μL (50 ng) cDNA, 1.5 μL RNAse-free water (Life Technologies, Carlsbad, CA, USA), 5 μL TaqMan Universal MasterMix II (Life Technologies, USA), and 0.5 μL TaqMan assays (Life Technologies, USA; listed in Table 1). All PCR runs were performed as described previously [45]. Data from the Real-Time PCR were normalized using the ratio of mRNA compared to the β-actin mRNA. The quantification of gene expression was performed using the comparative C.T. method.

### 4.9. Statistical Analysis

Statistical analyses were performed using GraphPad Prism 9.0 (GraphPad Software 2021, Inc., San Diego, CA, USA). For the determination of the results of all experiments, a one-way ANOVA followed by Dunnett’s post hoc test was used. All numerical data are presented as mean with standard deviation (SD), and differences are considered as statistically significant at the 95% confidence level (*p* < 0.05).

## Figures and Tables

**Table 1:** List of genes used.

Target Gene	Taq Man Sond Mouse	Taq Man Sond Swine
*CTSB*	Mm00514443	Ss03385626
*NFAT5*	Mm01247386	Ss06937542
*ATG7*	Mm00512209	Ss04248733
*ATG14*	Mm00553733	APH6FX7
*Casp3*	Mm01195085	Ss03382792
*Casp7*	Mm00432322	Ss06867774
*Casp9*	Mm00516563	Ss06438845
*BCL2*	Mm00477631	Ss0337516
*APAF1*	Mm01223701	Ss06439283
*MAPK1*	Mm00435945	Ss03821039
*MAPK2*	Mm00442498	APKCAH4
*Ki67*	Mm01278617	Ss06869821
*BECN1*	Mm01265461	Ss03380214
*ACTB*	Mm02619580	Ss03376563
*GAPDH*	Mm01180221	Ss03375629

## Data Availability

The datasets used and/or analyzed during the current study are available from the corresponding author on reasonable request.
